# Association between insulin resistance indices and kidney stones: results from the 2015–2018 National Health and Nutrition Examination Survey

**DOI:** 10.3389/fnut.2024.1444049

**Published:** 2024-10-02

**Authors:** Yue Shen, Zhu Zhu, Xiao Bi, Yuqi Shen, Aiwen Shen, Bo Deng, Yining He, Wenji Wang, Feng Ding

**Affiliations:** ^1^Department of Nephrology, Shanghai Ninth People's Hospital, School of Medicine, Shanghai Jiao Tong University, Shanghai, China; ^2^Department of Geriatrics, Shanghai Ninth People's Hospital, School of Medicine, Shanghai Jiao Tong University, Shanghai, China; ^3^Biostatistics Office of Clinical Research Unit, Shanghai Ninth People's Hospital, School of Medicine, Shanghai Jiao Tong University, Shanghai, China

**Keywords:** insulin resistance, kidney stone, METS-IR, TyG-BMI, population-based study

## Abstract

**Objective:**

To explore the association between representative insulin resistance (IR) indices and the risk of kidney stone disease in an American adult population. The representative IR indices referred to metabolic score for IR (METS-IR), triglyceride to high-density lipoprotein cholesterol (TG/HDL-C) ratio, triglyceride glucose-body mass index (TyG-BMI), visceral adiposity index (VAI), and homeostatic model assessment of IR (HOMA-IR).

**Methods:**

We investigated adult participants who joined the 2015–2018 National Health and Nutrition Examination Survey (NHANES) and reported kidney stone histories. Weighted proportions, multivariable regression analysis, and restricted cubic splines were used to evaluate the associations between IR indices and kidney stones after their adjustment for gender, age, race, education, smoking status, alcohol drinking frequency, hypertension and diabetes status, physical activity level, water intake, and levels of calcium, cholesterol, and uric acid.

**Results:**

A total of 19,225 participants were included. The weighted prevalence of kidney stone was 11.1%. A multivariable logistic regression model showed a dose–response relationship between the METS-IR and kidney stone [odds ratio (OR) = 1.02, 95% confidence interval (CI) (1.01, 1.04), *p* < 0.01]. A similar relationship was observed between the TyG-BMI and kidney stone after full adjustment [OR = 1.0, 95% CI (1.0, 1.01), *p* < 0.001]. Sex-stratified analyses revealed that the association between METS-IR and nephrolithiasis [OR = 1.03, 95% CI (1.01, 1.05), *p* < 0.01], and the association between TyG-BMI and nephrolithiasis [OR = 1.01, 95% CI (1.0, 1.01), *p* <0.001] was significant among the male participants in the fully adjusted model. Moreover, a significant association was found between the METS-IR levels and nephrolithiasis [OR = 1.03, 95% CI (1.01, 1.06), *p* < 0.01], and between the TyG-BMI levels and nephrolithiasis [OR = 1.01, 95% CI (1.0, 1.01), *p* < 0.05] among the diabetic participants after full adjustment. Furthermore, a potential nonlinear association was found between other IR indices (i.e., TG/HDL-C, VAI, and HOMA-IR) and the risk of kidney stone disease.

**Conclusion:**

Higher METS-IR and TyG-BMI levels were associated with a higher risk of nephrolithiasis. Future investigations are required to identify the role of IR in the progress of kidney stone formation and to propose prevention measures and health guidelines.

## 1 Introduction

Nephrolithiasis, also known as kidney stone disease, is caused by abnormal precipitation of urinary solutes and formation of crystalline substances in kidney (1). It affects approximately 10% of the global population and has shown an increasing tendency (2). Nephrolithiasis may lead to renal colic, infections, and chronic kidney disease, and thus, increases morbidity and hospitalizations (3, 4). To reduce morbidity and financial burden, research focused on the risk factors of kidney stone formation is required.

Nephrolithiasis is now recognized as a systemic and metabolic complication (1). Although the pathogenesis of urological stone disease is not fully understood, insulin resistance (IR) has long been speculated as a risk factor for urological stone disease (5, 6). As a prominent feature of obesity, prediabetes, and type 2 diabetes, IR alters ammoniagenesis by the renal tubules and affects systemic levels of inflammation and oxidative stress (7). Nephrolithiasis and IR are associated with overlapping pathogenic contributors, such as unhealthy dietary habits, lack of physical exercise, and abdominal obesity (8, 9). Evidence suggests that IR may facilitate stone formation by decreasing urine pH and altering urine composition (10). Urological stone disease is now recognized to be closely related to obesity and diabetes (11). Naturally, the relationship between indicators of IR and nephrolithiasis should be explored. Homeostasis model assessment of IR (HOMA-IR) (12), the triglyceride to high- density lipoprotein cholesterol (TG/HDL-C) ratio (13), and the metabolic score for IR (METS-IR) (14), have been shown to be effective measures for evaluating severity of IR. In particular, METS-IR, a reliable and predictive IR indicator proposed in 2018, was found to be associated with the occurrence of kidney stones (14, 15). In addition, the visceral adiposity index (VAI) is a gender-specific metabolic index that indirectly estimates visceral adipose and IR (16). A significant relationship was found between nephrolithiasis and VAI in patients who had undergone retrograde intrarenal surgery or percutaneous nephrolithotomy for kidney stones (17). Current research on the association between IR indices and nephrolithiasis is based on different populations in different research periods. Moreover, the association between nephrolithiasis and other IR indices, such as the triglyceride glucose-body mass index (TyG-BMI) and the TG/HDL-C ratio, has not yet been properly assessed. Here, we hypothesize that IR levels may be associated with the risk of kidney stone disease in the U.S. adults. This cross-sectional study aimed to investigate the association between five representative IR indices (i.e., TG/HDL-C, METS-IR, TyG-BMI, VAI, and HOMA-IR) and the risk of kidney stone disease from the National Health and Nutrition Examination Survey (NHANES) between 2015 and 2018.

## 2 Methods

### 2.1 Study participants

**Figure 1:**
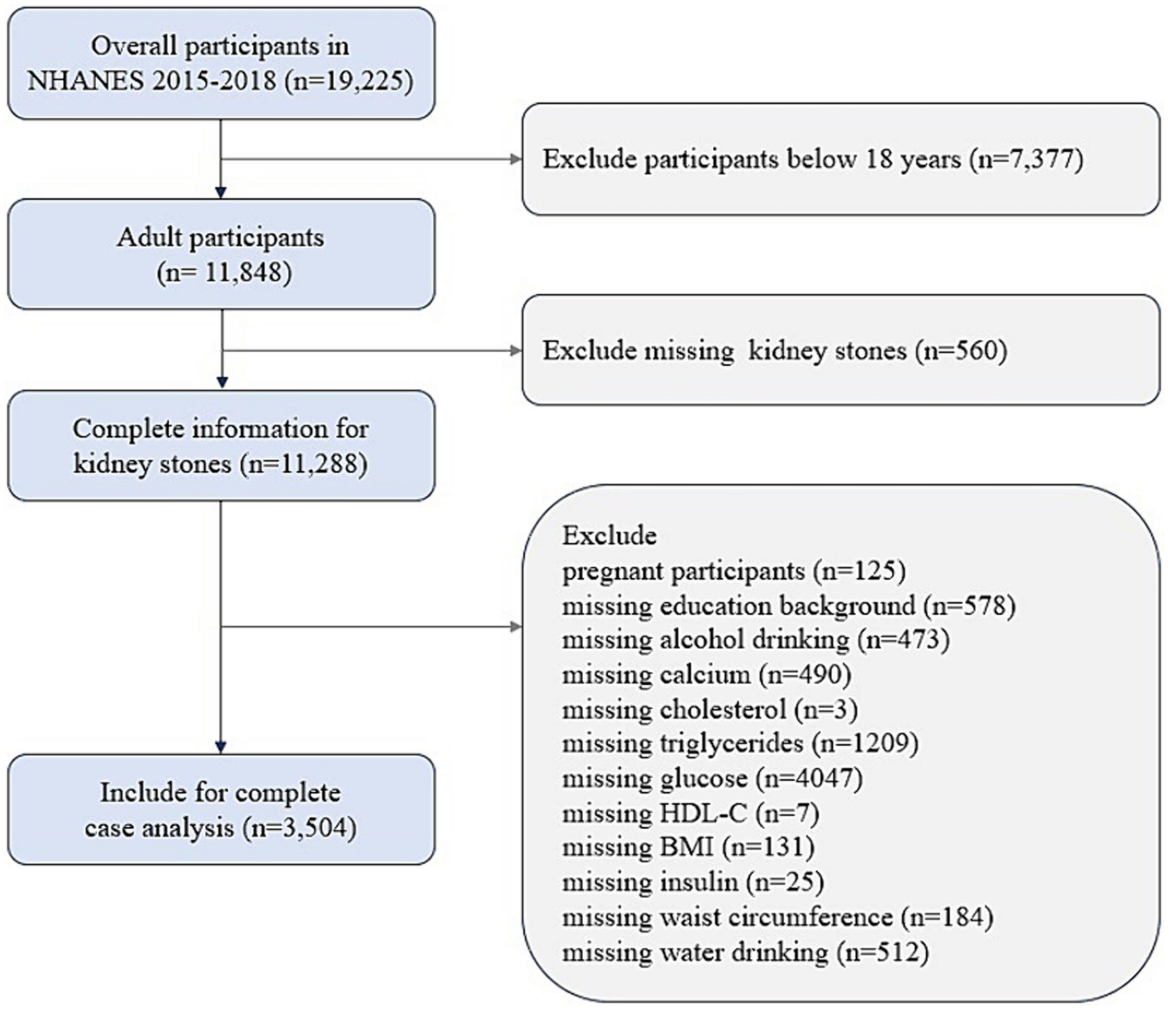
Flowchart of participants screening.

NHANES, which is conducted by the National Center for Health Statistics (NCHS), uses a complex sampling frame which obtains representative of the entire U.S. population and adjusts for the probable selection bias in the survey (18). The study protocols were approved by the NCHS institutional review board, and written consent was acquired from all participants. NHANES methodology and data collection have been fully described on the NHANES website.^1^ In the present study, cross-sectional data were obtained from 19,225 participants over two survey cycles spanning 2015–2018 in NHANES. Participants over the age of 20 with complete data on the IR indices and renal stones were available. The individuals with missing data on measures of alcohol and water drinking, education, BMI, waist circumference (WC), TG, fasting blood-glucose, HDL-C, fasting insulin, calcium, cholesterol, or pregnant females were excluded from the analysis. A total of 3,504 participants were included in the final analytic sample pool (Figure 1).

### 2.2 Measurements

#### 2.2.1 Outcome

The history of kidney stone was judged by “Ever had kidney stones?” Those who reported an answer to the question suggested diagnosed history of kidney stones. The data were considered missing for participants whose response was “Do not know” or “Refused.”

#### 2.2.2 Exposure

TG/HDL-C ratio was defined as TG (mg/dL) divided by HDL-C (mg/dL) (13, 19). METS-IR was calculated as Ln [2 × fasting glucose (mg/dL) + fasting TG (mg/dL)] × BMI / Ln (HDL-C) (mg/dL) (20). TyG-BMI = Ln [TG (mg/dL) × fasting glucose (mg/dL) / 2] × BMI (21). VAI, a lipid- based IR marker, is gender specific and was calculated as follows (22). For males: VAI = [WC (cm) / 39.68 + (1.88 × BMI)] × [(TG (mmol/L) /1.03)] × [1.31 / HDL-C (mmol/L)]. For females: VAI = [WC / 36.58 + (1.89 × BMI)] × (TG / 0.81) × (1.52 / HDL-C). HOMA-IR was defined as fasting glucose (mg/dL) × fasting insulin (mU/L) / 405 (23). BMI (kg/m^2^) was calculated as weight divided by height square.

#### 2.2.3 Covariates

Demographic information on sex (male/female), age (continuous; years), race/ethnicity (Mexican American, other Hispanic, Non-Hispanic White, Non-Hispanic Black, or other), education level (less than high school, equal to high school graduate, beyond high school) was obtained by interview questionnaires. In terms of the history of cigarette smoking (current, ever), current smoking patients refer to those who are currently smoking cigarettes and having smoked at least 100 cigarettes in one’s lifetime. As for alcohol consumption frequency, the participants were categorized into less than twelve drinks per year or at least twelve drinks per year. Hypertension (yes/no) was defined according to self-reported previous clinical diagnosis, or measurement of systolic blood pressure ≥ 140 mmHg or diastolic blood pressure ≥ 90 mmHg (24). Diabetes mellitus was defined as a previous diabetes diagnosis, or fasting plasma glucose ≥7.0 mmol/L, or glycohemoglobin ≥6.5% (25). All included individuals had two round of 24 h dietary recalls and the average water intake amount of the two recalls has been used in our study. The water intake amount was then categorized into tertiles, with the lowest tertile serving as a reference. The physical activity strength (vigorous or moderate or below moderate) was included in the adjusted multivariate model.

The levels of serum TG and HDL-C were detected by Cobas 6,000 Chemistry Analyzer. The levels of serum fasting glucose and fasting insulin were tested by Cobas C Chemistry Analyzer and Tosoh Bioscience AIA-900, respectively. Assayed by Roche Cobas 6,000 Chemistry Analyzer, the levels of serum calcium (mg/dL), cholesterol (mg/dL), and uric acid (mg/dL) were also identified as covariates that might influence the association between IR indices and kidney stones. There were no changes in the equipment or methods during the 2015–2018 cycles.

### 2.3 Statistical analysis

Data analyses were performed using IBM SPSS Statistics (version 22) and R 4.3.0. A two-tailed *p* value <0.05 was considered statistically significant. The statistical procedures were conducted according to the NCHC tutorials.^2^ A combination of Mobile Examination Center exam weights, fasting subsample weights and dietary sample weights was used based on the principle of using the smallest subpopulation weight of NHANES (26). The four-year weights were created for the 2015–2018 cycles by dividing the 2-year weights by 2. Continuous variables were described as mean ± standard error (SE), and categorical variables were summarized as percentages. The difference between subjects grouped was evaluated by a weighted Student’s *t*-test (for continuous variables) or weighted Chi-Square test (for categorical variables). As for the cross-sectional investigation, an adjusted multivariate logistic regression was applied to attain the association of IR indices and nephrolithiasis with summary of potential confounders (27). Apart from the demographic covariates (age, sex and race in Model 1), Model 2 included confounders of education, smoking and alcohol use, status of chronic diseases (diabetes and hypertension), water intake and physical activities. Water intake and physical activity have been suggested to reduce the occurrence and recurrence risk of nephrolithiasis (28, 29). Model 3 included variables in Model 2 plus serum levels of calcium, cholesterol, and uric acid, as hypercalcemia, hypercalciuria, dyslipidemia, and hyperuricemia have been regarded as the risk factors for urinary stone formation (30–33). Subgroup analysis and interaction tests were conducted in different gender and diabetic status. Additionally, restricted cubic spline (RCS) models with four knots were applied to explore the nonlinear association between the IR indices and nephrolithiasis. Associations were presented as predicted mean values and 95% confidence intervals (CI) of odds ratio (OR).

## 3 Results

### 3.1 Participant characteristics

The demographic and social characteristics and the laboratory results of the participants are presented in Table 1. Among the 3,504 participants, 371 had kidney stones. After weighting, approximately 11.1% (95% CI, 9.5–13.0%) of the sampled participants had kidney stones. These participants were senior compared to the participants without kidney stones (*p* < 0.05, Table 1). Whether or not diagnosed as kidney stones varied by race. Higher rates of hypertension and diabetes were found in the participants with nephrolith (*p* < 0.05). No significant difference was observed between the non-stone formers and the stone formers in gender, education, smoking status, alcohol drinking frequency, strength of work and recreational activities, amount of water intake, total calcium, total cholesterol, and uric acid level. Higher levels of the TG/HDL-C ratio, METS-IR, TyG-BMI, VAI, and HOMA-IR were observed in the participants diagnosed with nephrolithiasis (*p* < 0.05).

**Table 1:** Weighted demographic and clinical characteristics of the participants according to whether diagnosed as kidney stones or not.

	Non-stone former	Stone former	*p*
	(*N* = 3,133)	(*N* = 371)	
**Age, y**	47.4 ± 0.5	52.5 ± 1.2	<0.001^*^
**Female, %**	51.2	43.9	0.05
**Race, %**			0.002^*^
Mexican American	8.9	6.9	
Other Hispanic	6.6	6.5	
Non-Hispanic White	64.5	72.5	
Non-Hispanic Black	10.8	4.9	
Other Race	9.3	9.2	
**Education level, %**			0.248
Less than high school	11.0	12.7	
High school	25.0	19.2	
College	64.0	68.1	
**Hypertension, %**	37.8	51.1	0.001^*^
**Diabetes, %**	13.8	26.6	<0.001^*^
**Current smoker, %**	16.9	19.2	0.367
**Alcohol drinking**			0.989
Less than 12 times per year	34.2	34.3	
At least 12 times per year	65.8	65.7	
**Physical Activity**			0.544
Vigorous	47.2	47.9	
Moderate	31.4	27.2	
Below moderate	21.4	24.8	
**Water drinking**			0.927
1st tertile	30.2	30.5	
2nd tertile	33.0	31.3	
3rd tertile	36.8	38.2	
**Total Calcium, mg/dL**	9.3 ± 0.0	9.3 ± 0.0	0.125
**Cholesterol, mg/dL**	191.4 ± 1.3	189.8 ± 3.0	0.632
**Uric acid, mg/dL**	5.5 ± 0.0	5.7 ± 0.1	0.099
**TG/HDL-C**	2.7 ± 0.1	3.3 ± 0.2	0.046^*^
**METS-IR**	43.4 ± 0.5	48.1 ± 0.7	<0.001^*^
**TyG-BMI**	257.1 ± 2.4	278.7 ± 4.1	<0.001^*^
**HOMA-IR**	3.8 ± 0.1	5.0 ± 0.3	0.003^*^
**VAI**	2.0 ± 0.1	2.4 ± 0.2	0.047^*^

Continuous data are shown as mean ± SE and categorical data as percentage. *p* was calculated by regression test. *p <0.05.

### 3.2 Adjusted association between METS-IR and nephrolithiasis

The association between the METS-IR index and the risk of nephrolithiasis is presented in Table 2. METS-IR was positively associated with the OR of kidney stone disease after adjustment for age, sex, and race [1.03, 95% CI (1.02, 1.04), *p* < 0.001; 1SD increment: 1.02, 95% CI (1.01, 1.03), *p* < 0.001]. This association remained significant after further adjustments for education level, smoking status, alcohol consumption, diabetes and hypertension status, physical activity level, water intake, and levels of serum calcium, cholesterol, and uric acid [1.02, 95% CI (1.01, 1.04), *p* = 0.001; 1SD increment: 1.35, 95% CI (1.15, 1.58), *p* = 0.001], indicating that a unit increase in METS-IR index was associated with a 2% increase in the risk of nephrolithiasis. Furthermore, the METS-IR quartiles were associated with an increased risk of nephrolithiasis after adjustment for the covariates in different models. The OR of nephrolithiasis in the highest METS-IR quartile (Q4) was 1.77 [95% CI (1.2, 2.6), *P*_for trend_ = 0.002], 2.36 [95% CI (1.55, 3.61), *P*_for trend_ = 0.004] times greater than that in the lowest exposure quartile (Q1) after adjustment for the confounders in Model 2 and Model 3, respectively. RCS was employed to further explore if there was a nonlinear relationship between the METS-IR index and the risk of kidney stone disease. These results showed that there was no nonlinear relationship between the METS-IR index and nephrolithiasis (*P*_for nonlinearity_ = 0.65).

**Table 2:** Multivariable adjusted associations between METS-IR, TyG-BMI and nephrolithiasis.

	Nephrolithiasis OR (95% CI)		
	Model 1	Model 2	Model 3
METS-IR	1.03 (1.02, 1.04)^*^	1.02 (1.01, 1.04)^*^	1.02 (1.01, 1.04)^*^
METS-IR Q1 (ref)			
METS-IR Q2	1.63 (1.0, 2.64)^*^	1.92 (1.28, 2.86)^*^	2.72 (1.77, 4.19)^*^
METS-IR Q3	1.58 (0.96, 2.61)	1.8 (1.21, 2.66)^*^	2.46 (1.61, 3.76)^*^
METS-IR Q4	1.56 (0.95, 2.57)	1.77 (1.2, 2.6)^*^	2.36 (1.55, 3.61)^*^
*P*_for trend_	0.001	0.002	0.004
1SD increment of METS-IR	1.02 (1.01, 1.03)^*^	1.36 (1.19, 1.56)^*^	1.35 (1.15, 1.58)^*^

	Model 1	Model 2	Model 3
TyG-BMI	1.01 (1.0, 1.01)^*^	1.0 (1.0, 1.01)^*^	1.0 (1.0, 1.01)^*^
TyG-BMI Q1 (ref)			
TyG-BMI Q2	1.64 (1.06, 2.54)^*^	1.59 (1.03, 2.45)^*^	1.59 (1.03, 2.47)^*^
TyG-BMI Q3	1.56 (1.03, 2.35)^*^	1.49 (0.99, 2.24)	1.49 (0.99, 2.22)
TyG-BMI Q4	2.77 (1.75, 4.37)^*^	2.51 (1.6, 3.93)^*^	2.46 (1.51, 3.99)^*^
*P*_for trend_	0.000	0.000	0.000
1SD increment of TyG-BMI	1.39 (1.23, 1.58)^*^	1.35 (1.19, 1.55)^*^	1.34 (1.15, 1.57)^*^

Model 1 included terms for age (continuous), sex (female/male), and race (Mexican American, other Hispanic, Non-Hispanic White, non-Hispanic African, or other). Model 2 included terms for Model 1, education (less than high school, equal to high school graduate, beyond high school), current smoking (yes/no), alcohol consumption (less than twelve drinks per year or at least twelve drinks per year), diabetes (yes/no), hypertension (yes/no), water intake (low, median, high), and physical activity (vigorous, moderate or below moderate). Model 3 was further adjusted for terms in Model 2, calcium (continuous), cholesterol (continuous), and uric acid (continuous). ^*^ *p* < 0.05.

### 3.3 Adjusted association between TyG-BMI and nephrolithiasis

The association between the TyG-BMI index and the risk of nephrolithiasis is summarized in Table 2. The multivariable logistic regression analyses indicated that the TyG-BMI levels were positively associated with the risk of kidney stone disease after their adjustment for age, sex, and race [1.01, 95% CI (1.0, 1.01), *p* <0.001; 1SD increment: 1.39, 95% CI (1.23, 1.58), *p* < 0.001]. The association remained significant after further adjustments for the covariates in Model 3 [1.0, 95% CI (1.0, 1.01), *p* = 0.004; 1SD increment: 1.34 (1.15, 1.57), *p* < 0.001]. The OR of nephrolithiasis in the highest TyG-BMI quartile (Q4) was 2.46 [95% CI (1.51, 3.99), *P*_for trend_ <0.001] times greater than that in the lowest quartile (Q1) after adjustment for the confounders in Model 3. There was no nonlinear relationship between the TyG-BMI index and the risk of nephrolithiasis (*P*_for nonlinearity_ = 0.63), suggesting that the TyG-BMI index and kidney stone disease had a linear relationship.

### 3.4 Subgroup analysis for METS-IR and TyG-BMI

**Figure 2:**
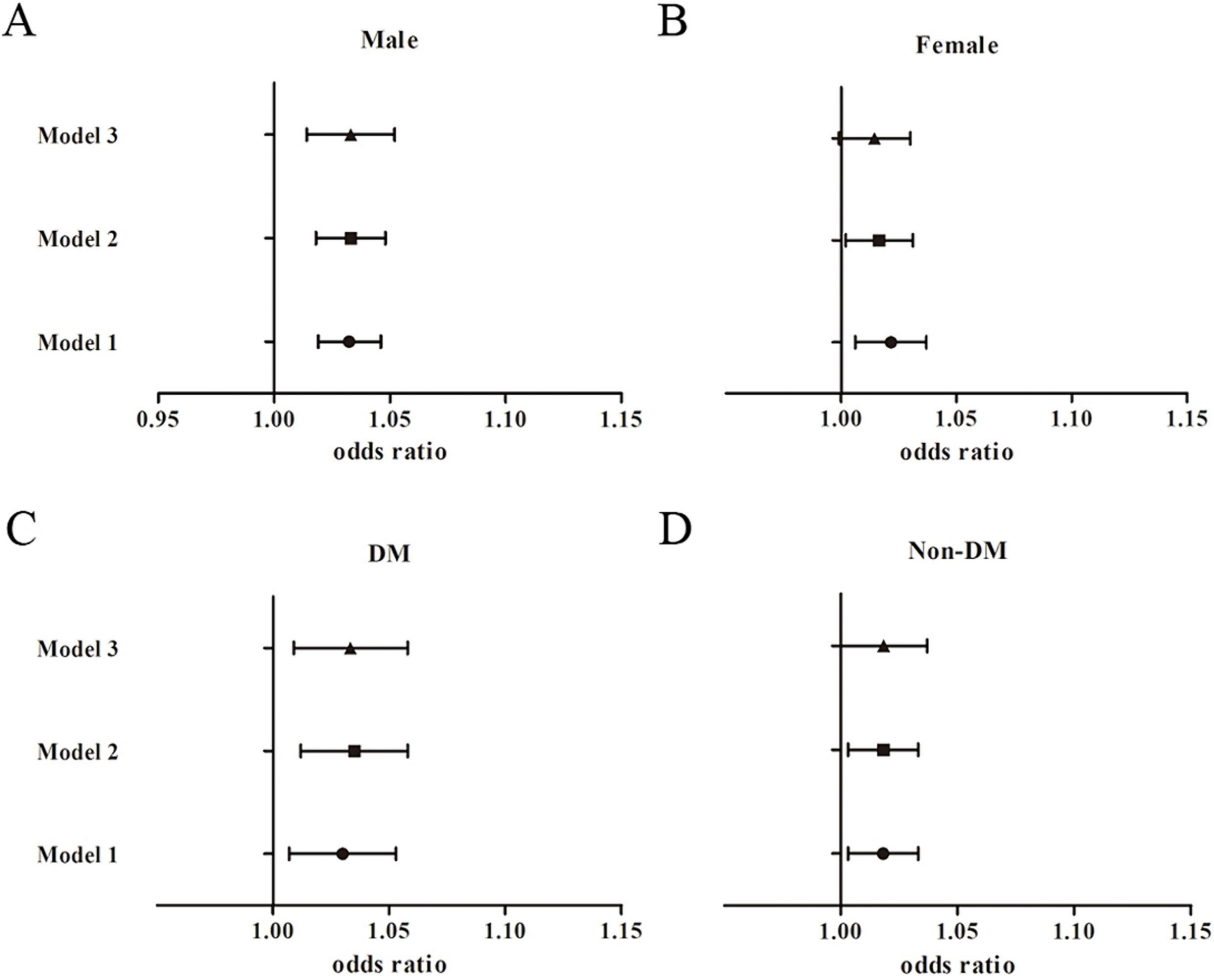
Subgroup analysis for the association between METS-IR and the risk of nephrolithiasis. **(A)** Male participants; **(B)** Female participants; **(C)** Diabetic participants; **(D)** Non-diabetic participants.

Sex-stratified analyses were performed to assess the robustness of the association between METS-IR and nephrolithiasis, and between TyG-BMI and nephrolithiasis. As Table 3 and Figure 2 show, the association between METS-IR and the risk of kidney stones was significant among the male participants [1.03, 95% CI (1.01, 1.05), *p* = 0.001] after adjustment for the covariates in Model 3. The OR of nephrolithiasis in the Q4 was 2.72 [95% CI (1.44, 5.14), *P*_for trend_ < 0.05] times greater than that in the reference quartile (Q1) after adjustment for the confounders in Model 3. The association between METS-IR and the risk of kidney stones was significant among the female participants [1.02, 95% CI (1.0, 1.03), *p* < 0.05] after adjustment for the covariates in Model 2. However, we did not observe a significant association between the METS-IR levels and the risk of kidney stones in the female participants [1.01, 95% CI (0.99, 1.03), *p* = 0.066; OR for Q4 vs. Q1: 1.99, 95% CI (0.97, 4.05), *P*_for trend_ = 0.2] after full adjustment. Among the participants with diabetes mellitus, a significant association was found between METS-IR levels and the risk of nephrolithiasis [1.03, 95% CI (1.01, 1.06), *p* = 0.009] by logistic regression after adjusting for covariates in Model 3 (Figure 2). Moreover, the OR of kidney stones in the highest METS-IR quartile was greater than that in the reference quartile (Q1) after adjustment for the confounders in Model 3 (*P*_for trend_ = 0.001). No significant association between METS-IR and the risk of kidney stones was perceived among the non-diabetic participants [1.02, 95% CI (0.99, 1.04), *p* = 0.05]. Interaction terms were also employed to test the heterogeneities in each subgroup, and the results showed no significant difference according to gender (*P*_for trend_ = 0.349) or diabetes status (*P*_for trend_ = 0.206), indicating that this positive association between METS-IR and the risk of kidney stone disease was not significantly influenced by sex or diabetic status.

**Table 3:** Subgroup analyses between METS-IR, TyG-BMI and nephrolithiasis.

	Nephrolithiasis OR (95% CI)				
METS-IR	Model 1	Model 2	Model 3	*P*_for trend_	*P*_for interaction_
**Stratified by gender**					
Female	1.02 (1.01, 1.04)^*^	1.02 (1.0, 1.03)^*^	1.01 (0.99, 1.03)	0.2	0.349
Male	1.03 (1.02, 1.05)^*^	1.03 (1.02, 1.05)^*^	1.03 (1.01, 1.05)^*^	0.027	
**Stratified by diabetes**					
No	1.02 (1.0, 1.03)^*^	1.02 (1.0, 1.03)^*^	1.02 (0.99, 1.04)	0.026	0.206
Yes	1.03 (1.01， 1.05)^*^	1.04 (1.01, 1.06)^*^	1.03 (1.01, 1.06)^*^	0.001	
**TyG-BMI**					
**Stratified by gender**					
Female	1.0 (1.0, 1.01)^*^	1.0 (1.0, 1.01) ^*^	1.0 (0.99, 1.01)	0.278	0.185
Male	1.01 (1.0, 1.01)^*^	1.01 (1.0, 1.01)^*^	1.01 (1.0, 1.01)^*^	0.012	
**Stratified by diabetes**					
No	1.0 (1.0, 1.01)^*^	1.0 (1.0, 1.01)^*^	1.0 (0.99, 1.01)	0.194	0.161
Yes	1.01 (1.0, 1.01)^*^	1.01 (1.0, 1.01)^*^	1.01 (1.0, 1.01)^*^	0.003	

Model 1 included terms for age (continuous), sex (female/male), and race (Mexican American, other Hispanic, Non-Hispanic White, non-Hispanic African, or other). Model 2 included terms for Model 1, education (less than high school, equal to high school graduate, beyond high school), current smoking (yes/no), alcohol consumption (less than twelve drinks per year or at least twelve drinks per year), diabetes (yes/no), hypertension (yes/no), water intake (low, median, high), and physical activity (vigorous, moderate or below moderate). Model 3 was further adjusted for terms in Model 2, calcium (continuous), cholesterol (continuous), and uric acid (continuous). ^*^ *p* < 0.05.

**Figure 3:**
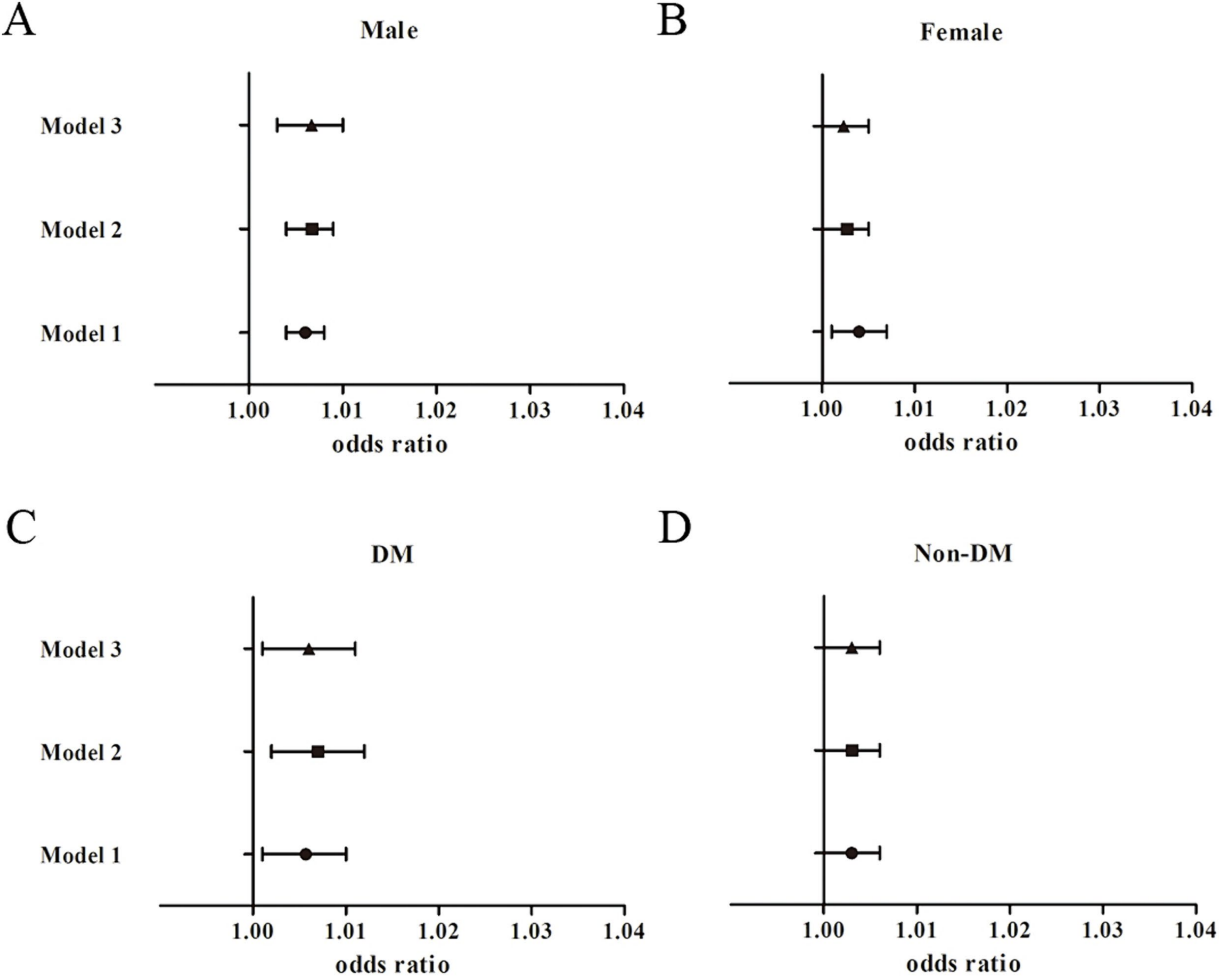
Subgroup analysis for the association between TyG-BMI and the risk of nephrolithiasis. **(A)** Male participants; **(B)** Female participants; **(C)** Diabetic participants; **(D)** Non-diabetic participants.

The subgroup analyses showed similar findings between TyG-BMI and nephrolithiasis (Figure 3). The association between TyG-BMI and kidney stones was significant among the male participants [1.01, 95% CI (1.0, 1.01), *p* < 0.001] after adjustment for the covariates in Model 3, and the OR of nephrolithiasis in the highest TyG-BMI quartile (Q4) was greater than that in the lowest quartile (Q1) [3.21, 95% CI (1.62, 6.34), *P*_for trend_ = 0.012]. The association between TyG-BMI and the risk of kidney stones was significant among the female participants [1.0, 95% CI (1.0, 1.01), *p* < 0.05] after adjustment for the covariates in Model 2. Yet we found no significant association between the TyG-BMI levels and nephrolithiasis risk in the female participants in the fully adjusted model [1.0, 95% CI (0.99, 1.01), *p* = 0.088; OR for Q4 vs. Q1: 2.07, 95% CI (1.01, 4.25), *P*_for trend_ = 0.278]. Among the diabetic participants, the association between TyG-BMI and kidney stones was significant [1.01, 95% CI (1.0, 1.01), *p* = 0.014]. The OR of kidney stones in the highest TyG-BMI quartile was greater than that in the reference quartile after adjustment for the confounders in Model 3 (*P*_for trend_ = 0.003). We found no significant association between TyG-BMI and the risk of kidney stones among the non-diabetic participants [1.0, 95% CI (0.99, 1.01), *p* = 0.052; OR for Q4 vs. Q1: 1.71, 95% CI (1.01, 2.89), *P*_for trend_ = 0.194]. Interaction terms were also used and the results showed no significant difference according to gender (*P*_for interaction_ = 0.185) and diabetes status (*P*_for interaction_ = 0.161).

In the prespecified subgroup analysis, a positive association was found between two IR indicators (i.e., METS-IR and TyG-BMI) and nephrolithiasis in the male and the diabetic participants. However, there was no significant interaction effect observed.

### 3.5 Association between other IR indices and nephrolithiasis

The association between other IR indices (i.e., the TG/HDL-C ratio, VAI index, and HOMA-IR index) and the risk of nephrolithiasis was also evaluated. The TG/HDL-C ratio [1.02, 95% CI (0.98, 1.05), *p* = 0.39], HOMA-IR [1.01, 95% CI (0.99, 1.02), *p* = 0.212], and VAI [1.03, 95% CI (0.97, 1.1), *p* = 0.304] were not linearly associated with the risk of nephrolithiasis after fully adjustment for the covariates in Model 3 (Supplementary Table S1).

**Figure 4:**
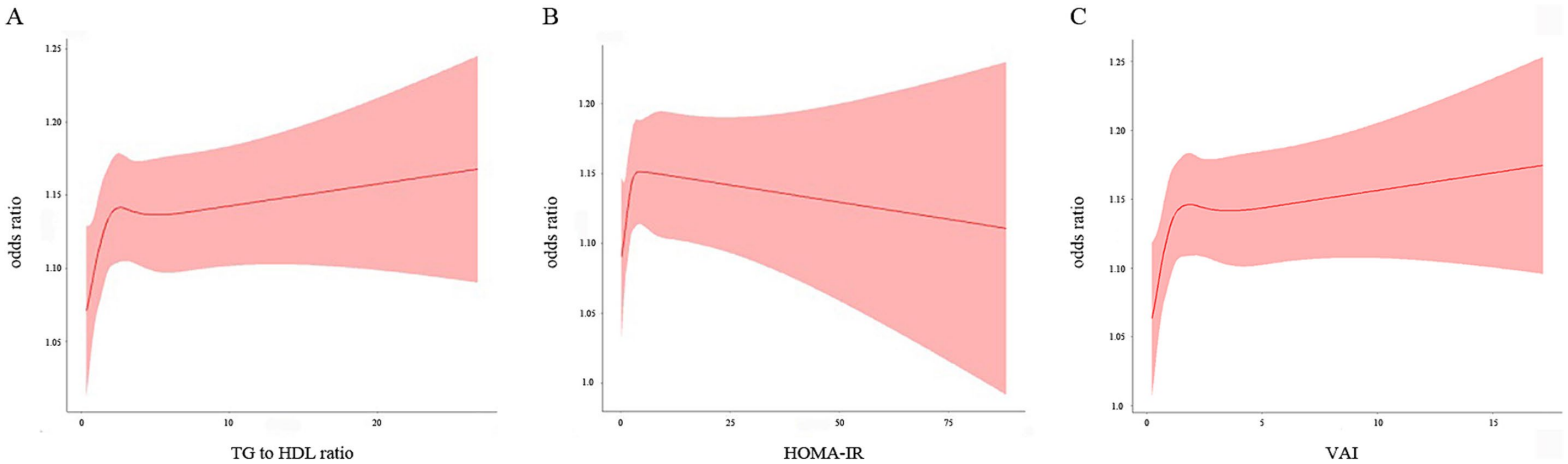
Restricted cubic spline plot of the association between three IR indices and the risk of nephrolithiasis. **(A)** TG/HDL-C; **(B)** HOMA-IR; **(C)** VAI. The associations were adjusted for gender, age, race, education, smoking, alcohol drinking, hypertension and diabetes status, physical activity, water drinking, calcium, cholesterol, and uric acid.

RCS analysis of the fully adjusted model revealed a nonlinear relationship between the TG/HDL-C ratio and the risk of kidney stone (*P*_nonlinear_ = 0.038). As shown in Figure 4A, the risk of kidney stone rapidly increased as the TG/HDL-C levels increased when the TG/HDL-C ratio was lower than 2.62, and slightly increased when the ratio was higher than 6.74. The RCS analysis for the HOMA-IR levels and the risk of kidney stone in the fully adjusted model (Figure 4B) showed a U-shaped association (*P*_nonlinear_ = 0.023) and presented the turning point of the RCS curve at approximately 4.26. The risk of kidney stone showed an ascending trend as the HOMA-IR levels increased when the index was below 4.26, and showed a decreasing trend when the index was above 4.26. VAI levels were also nonlinearly associated with the risk of nephrolithiasis (*P*_nonlinear_ = 0.014). As shown in Figure 4C, the risk of kidney stone presented a rapid increase trend as the VAI levels increased when the VAI was lower than 1.86, and showed a flatten increase trend when VAI was higher than 3.73.

### 3.6 Sensitivity analysis

BMI was incorporated into the formulas for three of the IR indices—TyG-BMI, METS-IR and VAI. Therefore, we tested the strength of the association between other IR indices (TG/HDL-C and HOMA-IR) and nephrolithiasis when BMI was added as a covariate in the logistic regression model. The association between TG/HDL-C [1.01, 95% CI (0.98, 1.05), *p* = 0.583], HOMA-IR [1.0, 95% CI (0.99, 1.06), *p* = 0.862] and the risk of nephrolithiasis did not considerably change when BMI was incorporated into the fully adjusted model. Also, the association between the VAI levels and the risk of nephrolithiasis did not alter [1.03, 95% CI (0.97, 1.1), *p* = 0.287] when gender was excluded from the models, since VAI is a sex-specific index.

## 4 Discussion

As stated in the introduction section, few studies have explored the association between various IR indices and the risk of kidney stone disease in one population and in one research period. In this study, we found significant associations between two IR indicators (METS-IR and TyG-BMI) and a history of kidney stones. The risk of kidney stones rose nonlinearly as the TG/HDL-C ratio and the VAI levels increased, and a U-shaped association between the HOMA-IR levels and the risk of kidney stones was found. These associations were largely preserved even after adjustment for various covariates. To sum up, METS-IR and TyG-BMI can serve as crucial indicators of nephrolithiasis risk. This finding suggests that fasting glucose, TG, and BMI might be involved in urinary stone formation. Therefore, the IR indices might have significant value for nephrolithiasis risk control and deserve further exploration in future studies.

As a common feature in metabolic syndrome and type 2 diabetes, IR refers to the diminished sensitivity or impaired response of the target organs or tissues to insulin (34, 35). The gold standard for quantifying IR is the hyperinsulinemic-euglycemic clamp technique. However, its wide-spread use is limited by its technical complexity and high cost (36). Alternative indices, such as the TyG-BMI (37), TG/HDL-C ratio (38), METS-IR (14), HOMA-IR (39), and VAI (23), have been shown to be practical and effective measures for evaluating IR. Previous findings suggested that poorer glycemic control and IR were associated with higher odds of kidney stone disease. Higher levels of TyG index have been reported to be associated with a higher risk of kidney stone disease in previous cross-sectional studies that did not include important confounders such as the amount of water intake, or the serum levels of calcium or uric acid (40, 41). In the present study, TyG-BMI, which is derived from the TyG index, proved effective in independently predicting nephrolithiasis. This research is the first to reveal the difference in the association between two IR indices (i.e., TyG-BMI and METS-IR) and nephrolithiasis by gender and diabetic status in the NHANES population. The findings of this study further suggest that male and diabetic patients are more vulnerable to kidney stone formation. Two other IR indices (TG/HDL-C ratio as an indicator of dyslipidemia and VAI as an indicator of visceral fat deposition) have also shown prediction strength for nephrolithiasis risk to a certain extent, which is in accordance with previous research (42, 43). With the rising rate of diabetes mellitus worldwide, the impact of IR on nephrolithiasis is likely to increase.

Nephrolithiasis is now recognized by the medical community as a chronic and complex medical condition which is associated to genetic factors, environmental factors (e.g., hot climate), chronic diseases (e.g., kidney disease and hyperparathyroidism), medication use, intestinal microbiome, among others (1, 44). The exact pathophysiologic mechanism underlying the role of IR in stone formation has not been fully elucidated yet. The change in urine composition due to diabetes and IR may serve as a major contributing factor (45). Most kidney stones are composed of oxalate calcium and phosphate calcium (46), and the pathophysiological mechanisms for calcium oxalate stone formation include low urine volume, hypercalciuria, hypocitraturia, hyperoxaluria, and hyperuricosuria (1). Thus, hypercalciuria (>0.1 mmol/kg per 24 h) is a significant risk factor of kidney stone formation (47). Previous studies have demonstrated increased urinary calcium, phosphorus, and oxalate excretion in diabetic patients (48, 49). Moreover, patients with dyslipidemia were found to have higher urinary calcium, and a better lipid profile would be beneficial for urine physicochemistry and stone risk (50). Antihyperlipidemic medicines (e.g., atorvastatin) increased urinary citrate, possibly by decreasing urinary uric acid and improving metabolic acidosis patients with calcium kidney stone (51). Studies have also suggested that uric acid excretion increases in the presence of hyperglycemia and glycosuria, which may account for the increased formation of uric acid stones among diabetic patients (52). *In vitro* and *in vivo* studies demonstrated that insulin stimulates renal ammonium production and excretion (7, 45, 53), hence IR may lead to defective renal ammonia excretion (54). Hyperglycemia and IR have also been proposed to impair hydrogen ion buffering, so urinary pH has also been regarded as a marker of renal insulin sensitivity (55). Diverse as the forming mechanisms of uric acid stone are, abnormally acidic urine is the principal factor (56). The status of IR increases kidney excretion of citrate, calcium, phosphorus, and uric acid (55, 57–59). As calcium and uric acid easily precipitate, patients with IR have increased risks of uric acid and calcium stone formation (60). Few studies have focused on the mechanisms of stone formation in cases of cystine and struvite stones, with the exception of evidence suggesting that low urinary pH caused by IR is a potential risk factor (1, 58). Improvement in peripheral IR therefore leads to an increase in urine pH and ammonia excretion and other changes in urinary composition that may retard stone formation. Treatment for IR (e.g., pioglitazone) has proved to increase renal ammonium excretion and result in higher urine pH (55).

This was a population-based study that involved standardized clinical and laboratory covariates. It has important implications for public health as well as clinical care because it shows that stone development may be prevented by IR alleviation and lifestyle modification. The results of this study prompt a better understanding of the role that metabolic syndrome plays in the pathophysiology of nephrolithiasis. Management strategies for kidney stones that target IR or the underlying pathobiological mechanisms are promising.

This study had limitations, including the inability to measure and analyze stone and urinary composition. Most kidney stones belong to the calcium oxalate type. However, the heterogeneity in the association between different types of stones and IR could not be neglected. Additionally, the nephrolithiasis data were based merely on patient self-reports, so recall or misclassification bias could have occurred. Self-reports may lead to bias on the associations in either way, potentially leading to false negative results among participants with fewer health issues (61). To our knowledge, no studies have assessed the reliability or validity of self-reported kidney stone in the U.S. population. Although a previous study indicated that the misclassification due to self-reported data was likely minimal (62), the conclusion drawn from our cross-sectional analysis should not be hastily generalized to whole population. The lack of imaging data suggested that all asymptomatic kidney stones could be excluded. The NHANES data after 2018 was not included in this study, because the data lacks completeness and representativeness due to the COVID-19 pandemic as explained on the NHANES website.^3^ Finally, because this study was not longitudinal, we could not determine the causal relationship between the IR indices and kidney stone development.

## 5 Conclusion

Kidney stones are common, painful, and costly. Higher IR indices (i.e., METS-IR, TyG-BMI, TG/HDL-C, and VAI) were found to be associated with a higher likelihood of kidney stone disease. These findings also suggest that kidney stones might be a systemic disorder, and in which case it could be utilized to optimize the risk stratification tools and health guidelines for preventing kidney stone development. Cohort studies that include individuals with varying levels of IR can provide valuable insights into establishing causal links between IR and nephrolithiasis. Interventional studies are recommended to examine the effects of IR alleviation on the risk of kidney stone development.

## Data Availability

The raw data supporting the conclusions of this article will be made available by the authors, without undue reservation.

## Footnotes

^1^ http://www.cdc.gov/nchs/nhanes.htm

^2^ https://wwwn.cdc.gov/nchs/nhanes/tutorials

^3^ https://wwwn.cdc.gov/nchs/nhanes/continuousnhanes/default.aspx?Cycle=2017-2020
