# Supplementary material for: Association between insulin resistance indices and kidney stones: results from the 2015–2018 National Health and Nutrition Examination Survey
Source: Front Nutr. 2024 Oct 2;11:1444049. doi: 10.3389/fnut.2024.1444049 (PMC11480067; doi:10.3389/fnut.2024.1444049)
Supplement: Supplementary file 1 [file Table_1.DOC]

**Table Supplementary 1.** Multivariable adjusted associations between TG/HDL-C ratio, HOMA-IR, VAI and the risk of nephrolithiasis.

|  | **Nephrolithiasis OR (95% CI)** | | ***P*_nonlinear_** |
| --- | --- | --- | --- |
|  | **Continuous** | **Q_4_ *vs.* Q_1_** |  |
| **TG/HDL-C** | 1.02 (0.98, 1.05) | 1.56 (0.77, 3.17) | .038 |
| Male | 1.0 (0.97, 1.04) | 2.02 (0.84, 4.86) |  |
| Female | 1.08 (0.95, 1.23) | 1.16 (0.49, 2.78) |  |
|  |  |  |  |
| **HOMA-IR** | 1.01 (0.99, 1.02) | 2.48 (1.53, 4.0) ^*^ | .023 |
| Male | 1.01 (0.99, 1.02) | 2.36 (1.27, 4.41) ^*^ |  |
| Female | 1.01 (0.99, 1.03) | 2.73 (1.31, 5.65) ^*^ |  |
|  |  |  |  |
| **VAI** | 1.03 (0.97, 1.1) | 1.64 (0.9, 2.99) | .014 |
| Male | 1.0 (0.98, 1.03) | 1.77 (0.75, 4.17) |  |
| Female | 1.04 (0.98, 1.11) | 1.25 (0.52, 2.98) |  |

*Note.* Model 1 included terms for age (continuous), sex (female/male), and race (Mexican American, other Hispanic, non-Hispanic white, non-Hispanic African, or other). Model 2 included terms for Model 1, education (less than high school, equal to high school graduate, beyond high school), current smoking (yes/no), alcohol consumption (less than twelve drinks per year or at least twelve drinks per year), diabetes (yes/no), hypertension (yes/no), water intake (low, median, high), and physical activity (vigorous, moderate or below moderate). Model 3 was further adjusted for terms in Model 2, calcium (continuous), cholesterol (continuous), and uric acid (continuous). ^*^*P* <.05.
